# Preoperative Systemic Therapy *Versus* Upfront Surgery in HER2-Positive Breast Cancer in the Real World

**DOI:** 10.3389/fonc.2021.704842

**Published:** 2021-07-28

**Authors:** Xingfei Yu, Chen Wang, Yabing Zheng, Beibei Miao, Jiejie Hu, Xiying Shao, Liming Sheng, Juan Lin, Yuqin Ding, Haojun Xuan, Yingying Ding, Lijie Gong, Weiliang Feng, Chengdong Qin, Daobao Chen, Yang Yu, Hongjian Yang

**Affiliations:** ^1^Department of Breast Tumor Surgery, Institute of Cancer Research and Basic Medical Sciences of Chinese Academy of Sciences, Cancer Hospital of University of Chinese Academy of Sciences, Zhejiang Cancer Hospital, Hangzhou, China; ^2^Department of Medical Oncology, Institute of Cancer Research and Basic Medical Sciences of Chinese Academy of Sciences, Cancer Hospital of University of Chinese Academy of Sciences, Zhejiang Cancer Hospital, Hangzhou, China; ^3^Taizhou Cancer Hospital, Taizhou, China; ^4^Department of Radiotherapy, Institute of Cancer Research and Basic Medical Sciences of Chinese Academy of Sciences, Cancer Hospital of University of Chinese Academy of Sciences, Zhejiang Cancer Hospital, Hangzhou, China

**Keywords:** human epidermal growth factor receptor 2, breast cancer, preoperative systemic treatment, surgery, real world

## Abstract

**Purpose:**

To compare survival in different strategies, preoperative systemic treatment *versus* upfront surgery, in HER2-positive early breast cancer patients in the real world.

**Methods:**

According to the actual upfront treatment, eligible patients from 2012 to 2015 were classified as preoperative systemic treatment or upfront surgery group prospectively. The primary endpoint is disease-free survival; the second endpoint is overall survival. All the outcomes were examined in the propensity score matching model and inverse probability of treatment weighting model.

**Results:**

Included in the analysis were 1,067 patients (215 in the preoperative systemic treatment group, 852 in the upfront surgery group). In the propensity score matching model (matching at 1:1 ratio), the disease-free survival of the preoperative systemic treatment group was significantly higher than that of the upfront surgery group (hazard ratio, 0.572, 95%CI, 0.371–0.881, *P*, 0.012). In the inverse probability of treatment weighting model, there was no significant difference in disease-free survival between the two groups (hazard ratio, 0.946, 95%CI, 0.763–1.172, *P*, 0.609). For overall survival, there was no significant difference between the two groups.

**Conclusion:**

The HER2-positive patients who accepted preoperative systemic treatment had better disease-free survival than those who underwent upfront surgery by real-world statistic methods.

**Clinical Trial Registration:**

Clinicaltrials.gov, identifier NCT04249440.

## Introduction

Preoperative systemic therapy (PST), mainly including neoadjuvant therapy for radical surgery and other systemic treatment before palliative surgery, is becoming increasingly popular in primary operable breast cancer (1). PST can allow for breast conservation surgery when upfront mastectomy is recommended; it also offers a better cosmetic result after the primary tumor shrinks. More importantly, PST enables identifying subgroups of patients with different prognoses: the patients with pathological complete response (pCR) after PST have better outcomes than those without pCR (2). It is prevalent in human epidermal growth factor receptor 2 (HER2)-overexpressing subtype for as high as 40–60% pCR rate of such a population (3). However, the National Surgical Adjuvant Breast and Bowel Project B-18 and B-27 trials from the USA have proved that PST could not improve either disease-free survival (DFS) or overall survival (OS) compared with upfront surgery (4–6). Several items should be noticed. First, in B18/27 trials, the ratio of HER2-positive cancer in the overall enrolled patients was not known. Second, in modern times, adding trastuzumab to chemotherapy significantly improved the DFS and OS in both neoadjuvant and adjuvant settings, but all the patients enrolled in B18/27 trials accepted only chemotherapy, which seems not sufficient. We designed a real-world study to investigate the prognosis of anti-HER2 treatment combined with chemotherapy preoperatively comparing with upfront surgery (US) in HER2-positive early breast cancer (NCT04249440).

## Materials and Methods

### Eligibility and Treatment Assignment

Patients diagnosed with HER2-positive early invasive breast cancer for the first time at Cancer Hospital of the University of Chinese Academy of Sciences were identified and enrolled continuously from January 2012 to January 2015. The investigators obtained informed consent from each participant or each guardian of the participant. Investigators performed the human investigations after approval by the Human Investigations Committee and the Department of Health and Human Services of Zhejiang Cancer Hospital. Reporting of the study conforms to broad EQUATOR guidelines.

The inclusion criteria included female, stage cT1-3N0-1M0 (AJCC 7th), HER2-positive expression in primary invasive tumor, accepted preoperative systemic treatment followed by surgery, or upfront surgery followed by adjuvant treatment. The conditions of HER2-positive expression of primary breast cancer were defined as follows: HER2 3+ by immunohistochemical (IHC) method or HER2 2+ by IHC with a further positive result by fluorescence *in situ* hybridization (FISH). Exclusion criteria were as follows: accepting any other anti-HER2 target drugs beyond trastuzumab (pertuzumab could not be acquired before 2018 in China); absence of chemotherapy besides anti-HER2 target drugs; the operation was performed more than one month after PST was accomplished; adjuvant chemotherapy started more than one month after surgery in upfront surgery group; trastuzumab use for less than one year; incomplete clinicopathologic data. According to the actual upfront treatment, all eligible patients were classified as either PST or upfront surgery (US) groups. All the patients should accept chemotherapy (anthracyclines, taxanes, cyclophosphamide, or carboplatin as main drugs) and standard one-year trastuzumab as anti-HER2 treatment besides chemotherapy. In the PST group, the effect was evaluated according to RECIST 1.1 every two cycles. The patients with clinical complete response (cCR) or partial response (PR) would receive the whole course of chemotherapy and trastuzumab. Those with stable disease (SD) or progressive disease (PD) would receive surgery promptly. After surgery, all patients underwent irradiation and endocrine therapies if necessary. The pCR was defined as the absence of infiltrating residual invasive disease in the breast and axillary nodes. For those non-pCR patients after PST, intensive adjuvant chemotherapy was not routine treatment except for PD patients.

The data elements include the age of the patient age at diagnosis, clinical staging (T, N, AJCC 7th) at diagnosis, histologic tumor grade, estrogen receptor (ER), and progesterone receptor (PR) expressions of the primary tumor by IHC. The accreditation requires an annual 95% follow-up rate for all eligible patients diagnosed within five years.

### Outcome Measures

The primary endpoint was DFS, defined as the time from enrollment to local, regional, or distant recurrences; the occurrence of contralateral breast cancer; or death without evidence of breast cancer. Patients evaluated as PD was thought to be local treatment failures. The second endpoint was OS, defined as the time from study entry to death from any cause. All the endpoints were compared between the PST group and the US group. Also, further analysis according to pathological response stratified in the PST group was performed.

### Statistical Methodology

All the data were collected and analyzed by SPSS (v 26.0, IBM Corp almonk, NY, USA). The categorical data were analyzed by the Pearson’s χ^2^ test and Fisher’s exact test if necessary. We used the Wilcoxon rank sum test for ordinal and continuous data.

According to the real-world study statistical methods (7), we built a propensity score matching (PSM) model by matching average treatment effect for further controlling confounding factors. The match ratio of the two groups was 1:1, and match tolerance was 0.01, which could show satisfying match score comparability. All clinicopathologic parameters were included in the models for multivariate logistic regression in calculating the propensity score: age at diagnosis, clinical stage T/N at diagnosis, tumor histologic grade (grades 1 and 2 were analyzed together due to a meager percentage of HER2-positive breast cancer patients with grade 1 tumors), and ER/PR expression in the primary tumor by IHC (1% as the cut-off value of positive expression). Standardized mean differences were calculated to assess the equivalence between matched participants (PST group *vs*. US group).

The propensity score was calculated according to the regression results of all characteristics for the treatment strategy mentioned above. A stabilized inverse probability of treatment weight (IPTW) was calculated for all participants based on the propensity score. The group differences were assessed by calculating IPTW proportions and standardized mean differences. The distribution cone diagram showed a satisfying, balanced distribution of propensity scores in the two groups (**Figure 2**) in the PSM and IPTW models.

The survival curves were estimated by the Kaplan–Meier method, and PST *versus* US comparisons was made using the absolute difference of survival rate and relevant hazard ratio in unweighted, PSM, and IPTW models. The general data hypothesis test level was set at an alpha of 0.05 (double-tailed). Patients with missing values for an endpoint were not included in the endpoint analysis; missing data were not imputed.

## Results

**Figure 1:**
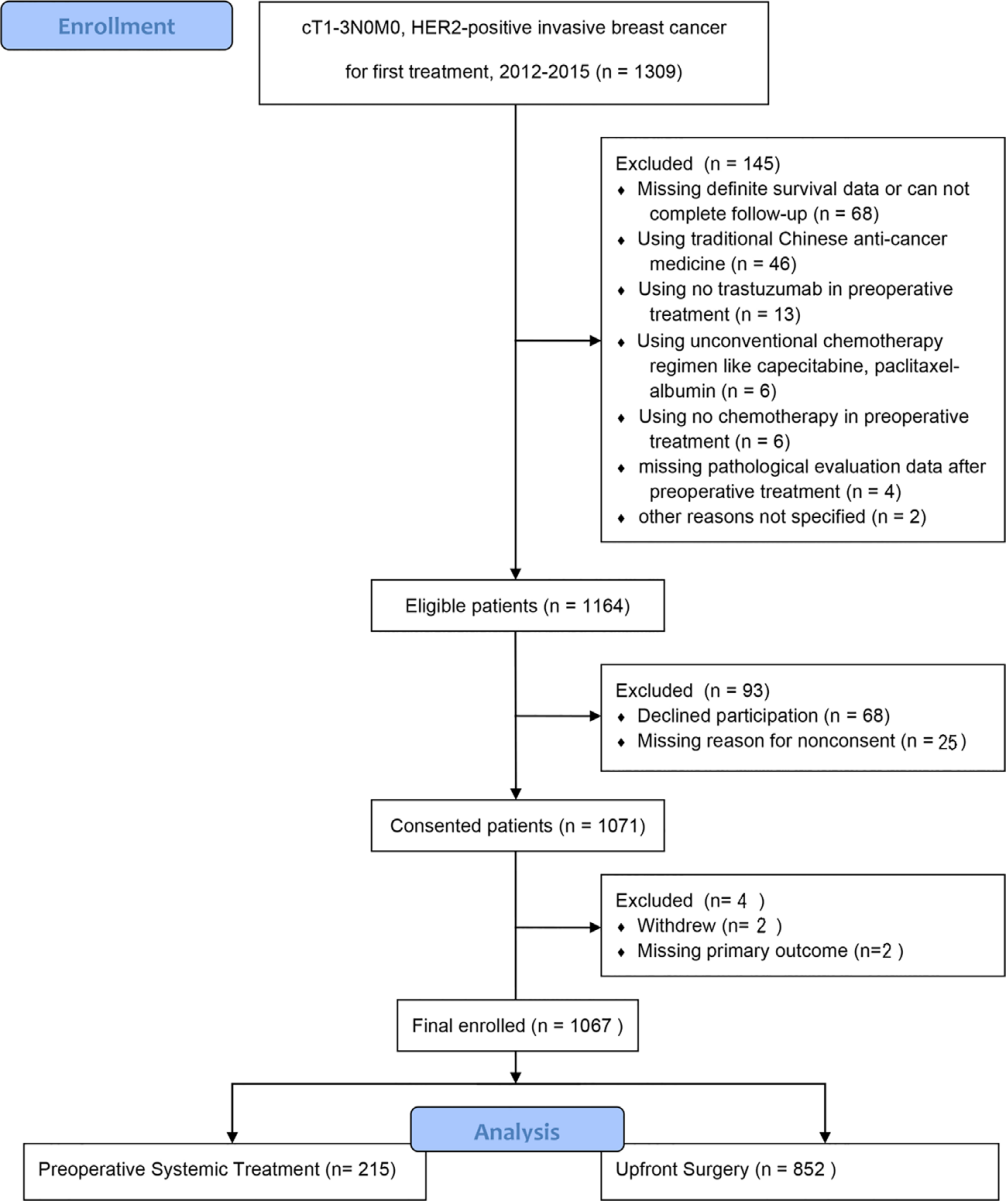
Flow diagram of participants with operable HER2-positive breast cancer.

In total, 1,164 of 1,309 (88.9%) patients completed the primary outcome assessment. Of these, 68 were excluded because of declined participation; 29 were excluded for other reasons. Finally, the analysis included 1,067 patients, of whom 215 underwent PST and 852 underwent US (**Figure 1**).

### Clinicopathologic Characteristics

Of the patients 215 patients (20.15%) accepted PST and 852 patients (79.85%) underwent upfront surgery. The median age was similar in the two groups (**Table 1**). The patients of the PST group had higher stage T/N, higher grade, and less positive expression of ER and PR than those of the US group in univariate analysis.

**Table 1:** The clinicopathologic characteristics of two groups.

		PST group (215)	US group (852)	Statistics of test
		N (%)	N (%)	
Age (years, median, 95%CI)		49, 35–65	49, 35–62	Z = 0.051, *P* = 0.951
Stage T	1	8 (3.7)	450 (52.8)	χ^2^ = 215.434, *P* < 0.001^*^
	2	157 (73.0)	372 (43.7)
	3	50 (23.3)	30 (3.5)
Stage N	0	37 (17.2)	534 (62.7)	χ^2^ = 142.663, *P* < 0.001
	1	178 (82.8)	318 (37.3)
Grade	1 and 2	92 (42.8)	430 (50.5)	χ^2^ = 4.051, *P* = 0.044
	3	123 (57.2)	422 (49.5)
ER	Negative	142 (66.0)	394 (46.2)	χ^2^ = 26.929, *P* < 0.001
	Positive	73 (34.0)	458 (53.8)
PR	Negative	171 (79.5)	478 (56.1)	χ^2^ = 39.557, *P* < 0.001
	Positive	44 (20.5)	374 (43.9)

^*^Fisher’s exact test. PST, preoperative systemic treatment; US, upfront surgery; ER, estrogen receptor; PR, progesterone receptor.

### The Effect of PST

In the PST group, 19.5 and 72.1% of cases had cCR and PR, respectively. Fifteen cases were evaluated as SD, and three cases had PD; they all had surgery without completing the whole course of neoadjuvant treatment. After surgery, 36.7% (75/215) of patients of the PST group achieved pCR.

### Outcomes in Unweighted Primary Sample

**Figure 2:**
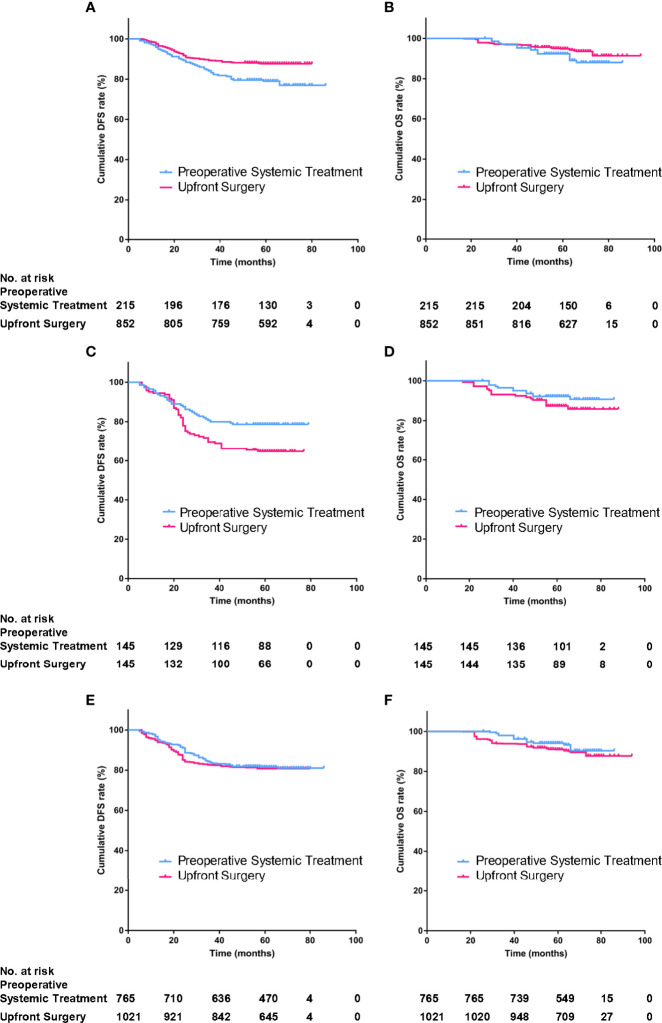
The DFS and OS of preoperative systemic treatment (PST) group and upfront surgery (US) group in primary unmatched model **(A, B)** in propensity score matching (PSM) model **(C, D)**, in inverse probability of treatment weighting (IPTW) model **(E, F)**.

**Figure 3:**
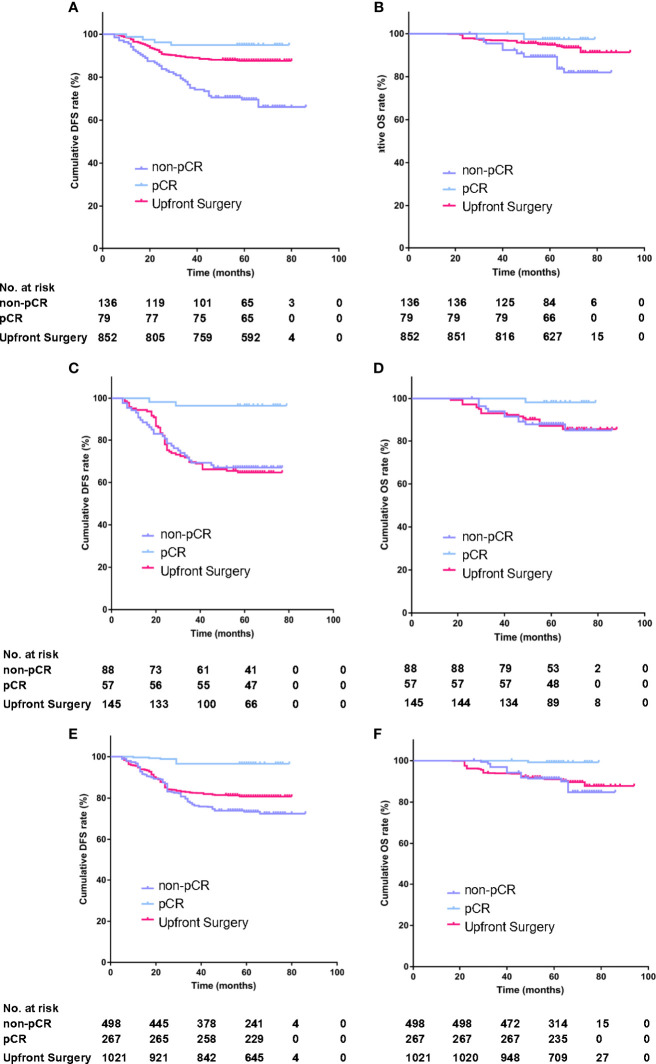
The stratified analysis of DFS and OS according to pathological response status in the PST group in primary unmatched model **(A, B)** in propensity score matching (PSM) model **(C, D)**, in inverse probability of treatment weighting (IPTW) model **(E, F)**.

By January 2020, the median follow-up time was 62 (95%CI, 17–76) months. There was a total of 152 events (14.2%); 76 patients (7.1%) died during the follow-up. There were 47 patients (21.9%) of the PST group and 105 patients (12.3%) of the US group who relapsed; 21 patients (10.0%) of the PST group and 55 patients (6.5%) of the US group died of different causes. By Kaplan–Meier method in unweighted primary sample (**Figures 2A, B** **)**, the cumulative DFS rate of the US group was 87.7%, significantly higher than the 78.1% of the PST group (HR = 1.828, 95%CI, 1.225–2.727, *P* = 0.0012, **Table 4**), the OS rate of the US group was 94.1%, similar to 90.2% of the PST group (HR = 1.607, 95%CI, 0.905–2.852, *P* = 0.064, **Table 4**). Multivariate analysis using Cox proportional hazards model indicated that age, stage T, stage N, ER, and PR had significant impacts on DFS; the upfront treatment had a weak influence on DFS, and age, stage T, stage N, and grade were significantly correlated with OS (**Table 2**). Further, in a stratified analysis of the pathological response status of the PST group, the patients who did not achieve pCR had significantly lower cumulative DFS and OS rates (68.4 and 86.0%) than those with pCR (94.9 and 97.5%) and those of the US group (87.7 and 94.1%) (**Figures 3A, B**, **Table 4**).

**Table 2:** Multivariate analysis in Cox proportional hazards model.

Unweighted Sample	DFS			OS		
	B	HR (95% CI)	p	B	HR (95% CI)	p
Age	−0.022	0.978 (0.961–0.996)	0.016	−0.043	0.958 (0.932–0.983)	0.001
Stage T	0.786	2.194 (1.624–2.968)	<0.001	0.653	1.922 (1.240–2.979)	0.003
Stage N	1.275	3.578 (2.351–5.444)	<0.001	0.660	1.934 (1.100–3.403)	0.022
Grade	0.115	1.122 (0.800–1.575)	0.504	−0.527	0.590 (0.358–0.974)	0.039
ER	1.076	2.933 (1.960–4.389)	<0.001	0.355	1.426 (0.764–2.660)	0.265
PR	−0.892	0.410 (0.264–0.636)	<0.001	−0.523	0.593 (0.295–1.192)	0.142
US (*vs* PST)	−0.363	0.696 (0.470–1.030)	0.070	−0.215	0.807 (0.451–1.442)	0.469

DFS, disease-free survival; OS, overall survival; HR, hazard ratio; ER, estrogen receptor; PR, progesterone receptor; US, upfront surgery; PST, preoperative systemic treatment.

### Outcomes in PSM Model and IPTW Model

**Figure 4:**
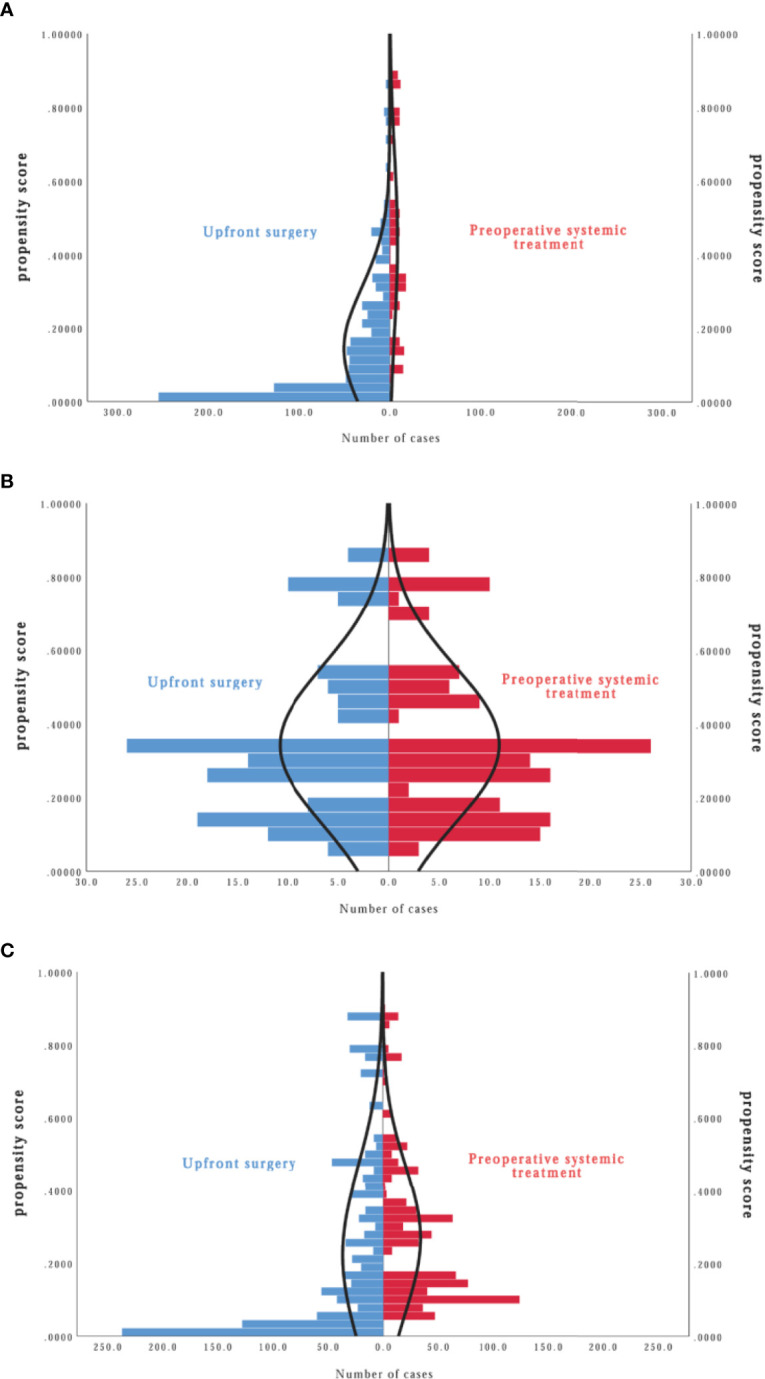
Distribution cone diagram of propensity score in two groups in unmatched model **(A)**, in propensity score matching (PSM) model **(B)**, in inverse probability of treatment weighting (IPTW) model **(C)**.

The propensity score was calculated according to the regression results of all characteristics for treatment strategy. The distribution cone diagram showed a satisfying balanced distribution of propensity score in two groups (**Figures 4A–C**) in PSM model and IPTW model.

In the PSM model, 145 cases in the PST group matched successfully with the US group at a 1:1 ratio by propensity score at ±0.01 of difference level. Patients of the PST group and the US group had no significant differences in age, stage T, stage N, histological grade, or PR expression after matching. Simultaneously, the PST group had less ER expression and higher grade than the US group (**Table 3**). The DFS rate of the PST group was 77.3%, significantly higher than 63.1% of the US group (**Figure 2C**, **Table 4**, HR, 0.572, 95%CI, 0.371–0.881, *P* = 0.012). There was no significant difference in OS rate between the two groups (**Figure 2D**). In further stratification analysis, the DFS rate of the patients with pCR after PST was 96.1%, higher than those without pCR and the US group (**Figures 3C, D** **)**.

**Table 3:** The clinicopathologic characteristics of two groups in PSM and IPTW models

Characteristics		Number of cases	Unweighted primary sample			PSM model			IPTW model^*^		
			PST group (215)	US group (852)	SMD	PST group (145)	US group (145)	SMD	PST group (765)	US group (1021)	SMD
			N (%)	N (%)		N (%)	N (%)		N (%)	N (%)	
Age (years, medium, 95%CI)			50, 39~61	50, 33~64	0.05	50, 40~65	49, 34~62	0.10	49, 39~67	50, 31~64	0.03
Stage T	1	458	8 (3.7)	450 (52.8)	0.701	8 (3.7)	10 (6.9)	0.032	80 (10.5)	450 (44.1)	0.467
	2	529	157 (73.0)	372 (43.7)		118 (81.4)	116 (80.0)		635 (83.0)	458 (44.9)	
	3	80	50 (23.3)	30 (3.5)		19 (13.1)	19 (13.1)		50 (6.5)	113 (11.1)	
Stage N	0	571	37 (17.2)	534 (62.7)	1.048	37 (17.2)	32 (22.1)	0.081	310 (40.5)	538 (52.7)	0.246
	1	596	178 (82.8)	318 (37.3)		108 (81.4)	113 (77.9)		455 (59.5)	483 (47.3)	
Grade	1 and 2	522	92 (42.8)	430 (50.5)	0.154	77 (53.1)	89 (61.4)	0.168	345 (45.1)	493 (48.3)	0.064
	3	545	123 (57.2)	422 (49.5)		68 (46.9)	56 (38.6)		420 (54.9)	528 (51.7)	
ER	Negative	536	142 (66.0)	394 (46.2)	0.407	87 (60.0)	68 (46.9)	0.265	386 (50.5)	513 (50.2)	0.004
	Positive	531	73 (34.0)	458 (53.8)		58 (40.0)	77 (53.1)		379 (49.5)	508 (49.8)	
PR	Negative	649	171 (79.5)	478 (56.1)	0.418	108 (74.5)	113 (77.9)	0.081	499 (65.2)	625 (61.2)	0.083
	Positive	418	44 (20.5)	374 (43.9)		37 (25.5)	32 (22.1)		266 (34.8)	396 (38.8)	

^*^Proportions and medians are weighted using IPTW, all covariates included in the propensity analysis. Abbreviations: PSM, propensity score matching; IPTW, inverse probability of treatment weighting; PST, preoperative systemic treatment; US, upfront surgery; SMD, standardized mean difference; ER, estrogen receptor; PR, progesterone receptor.

In the IPTW model, the patients of the PST group and the US group had no significant differences in age, histological grade, ER, or PR expression after matching (**Table 3**). The DFS rate of the PST group was 81.3 *versus* 80.8% of the US group, and the OS rate of the PST group was 92.1 *versus* 90.3% (**Figure 2**), both having no significant differences (**Table 4**). In a further stratification analysis (**Figures 3E, F** **)**, in the PSM model, the DFS and OS rates of the patients without pCR after PST (73.1 and 88.4%) were worse than those with pCR (96.6 and 99.3%) and US group (80.8 and 90.3%), respectively.

**Table 4:** Summary of results of the survival analysis.

Type analysis (HR: PST *vs* US)	N		DFS			OS		
	PST group	US group	Absolute difference (95%CI)	HR (95%CI)	*P*	Absolute difference (95%CI)	HR (95%CI)	*P*
Primary unweighted sample	215	852	—	—	—	—	—	—
pCR after PST	79		7.26% (1.95% ~12.58%)	0.527 (0.271 ~1.023)	0.059	3.34% (−0.47% ~7.14%)	0.377 (0.150 ~0.947)	0.155
Non ~pCR after PST	136		19.29% (11.17% ~27.41%)	2.776 (1.716 ~4.493)	<0.001	8.10% (0.07% ~14.14%)	2.998 (1.570 ~5.722)	<0.001
PSM model	145	145	13.79% (3.55% ~24.04%)	0.572 (0.371 ~0.881)	0.012	4.83% (−0.26% ~11.92%)	0.622 (0.307 ~1.257)	0.191
IPTW model	765	1021	0.50% (−3.17% ~4.17%)	0.946 (0.763 ~1.172)	0.609	1.85% (−0.78% ~4.49%)	0.740 (0.541 ~1.011)	0.062

HR, hazard ratio; DFS, disease ~free survival; OS, overall survival; PST, preoperative systemic treatment; US, upfront surgery; pCR, pathological complete response; PSM, propensity score matching; IPTW, inverse probability of treatment weighting.

## Discussion

Trastuzumab has been proven beneficial in the neoadjuvant setting. The main regimens included docetaxel, paclitaxel, anthracyclines, and carboplatin, showing a favorable toxicity profile. The pCR rates varied from 18 to 47% across the phase II studies, both in early and local advanced HER2-positive breast cancer (8–11). It had been demonstrated that HER2-positive breast cancer could achieve a higher pCR rate than other subtypes, and this short-term benefit could be transferred into long-term survival benefit (12). For this point, patients with HER2-positive breast cancer may get more benefit from PST compared with other molecular subtyping patients. Due to the deficiency of anti-HER2 treatment and HER2 subtyping, the NSABP B18/27 (National Surgical Adjuvant Breast and Bowel Project B-18 and B-27) trials from the USA could not precisely indicate that PST was similar to upfront surgery in the HER2-positive population on extended survival. After those two studies, fewer trials compared the survival of these two different strategies in HER2-positive early breast cancer, and now it is impracticable to run such a prospective randomized trial.

We designed a real-world study to investigate this problem. The results indicate that patients receiving upfront surgery have better survival than those receiving PST before adjusting the parameters. There may be several reasons. Firstly, in the real world, patients receiving upfront surgery usually have smaller tumor and less lymph node involved than those receiving PST. In our study, there were 52.8% of T1 and 62.7% of N0 in the US group *versus* 3.7% of T1 and 17.2% of N0 in the PST group. Secondly, over half of the patients of the upfront surgery group had ER-positive expression *versus* only nearly 1/3 of patients of the PST group, which means the former has lower clinical risk and benefit more from endocrine therapy.

To balance the characteristics and reduce the impact of confounding factors as much as possible, we further analyzed the survival data using the PSM and IPTW methods. The results indicate that in the 141 matching pairs from two groups, the patients of the PST group acquired significantly longer DFS and less recurrence than the ones of the US group. This is the first study to report that PST might improve survival in early HER2-positive breast cancer after balancing baseline characteristics from real-world data. Further analysis implies the difference of survival benefit mainly came from patients who achieved pCR after PST, while the patients of upfront surgery had a similar DFS with those who achieved non-pCR after PST.

In recent years, many studies support the use of dual-HER2 blockade for neoadjuvant treatment of HER2-positive breast cancer patients, regardless of whether lapatinib (13, 14) or pertuzumab was used as a second agent (15). Dual-HER2 blockade is superior to chemotherapy with trastuzumab in terms of higher pCR rate (16). Several trials report that the hormone receptor-negative groups responded better to chemotherapy combined with HER2 blockade therapy and achieved a better pCR rate (14, 17, 18). All these studies used the dual-HER2 blockade treatment in the neoadjuvant setting but not continued for the postoperative adjuvant phase in their protocols. In the adjuvant setting, the significant improvements in DFS is observed in chemotherapy combined with trastuzumab and pertuzumab in APHINITY study (19), especially for lymph node-involved patients, but not in chemotherapy combined with trastuzumab and lapatinib in ALTTO study (20). The PEONY study is the only one researching the dual-HER2 blockade treatment in neoadjuvant and lasts one year after surgery. The result should be noticed if the whole course of dual-target treatment would be suggested. So far, the consensus is that adding another HER2 blockade treatment to trastuzumab and chemotherapy preoperatively can increase pCR rate, but whether it improves survival, especially under the same postoperative treatment, is still controversial. Our study began in 2012 while pertuzumab was not available until 2019 in China. The patient enrolled accepted trastuzumab combined with chemotherapy as the standard treatment in the neoadjuvant and adjuvant setting groups. Even though it seems partly deficient in modern times, the two groups can still be comparable, and the result can be reasonable and trustworthy.

As we know now, the adjuvant T-DM1 treatment in residual disease settings can increase five year-DFS after PST (21). This survival benefit is confirmed in the non-pCR population, using single- or dual-HER2 blockade treatment during the PST phase. The homogeneity of benefit is seen across all subgroups. PST can practically guide the adjuvant strategies in changing the prognosis; PST is more than just giving bioinformation and treatment sensitivity. PST has the advantage of further improving survival; it might be more valuable than the US strategy, which cannot identify the high-risk patients.

Our study has several limitations. First, chemotherapy regimens are not predetermined and impractically balanced in two groups. The individualized choice of regimens in big data of the natural world is still a challenge for statistics. Second, as mentioned above, the anti-HER2 treatments included duel-HER2 blockade regimens. Third, propensity score matching is an ideal method to control confounding factors and balance clinicopathological characteristics in the real-world study, but this would weaken the power of tests. Our data finally show the significant difference in DFS between the two groups even under such a situation.

## Conclusion

Real-world study indicates that patients with HER2-positive breast cancer have a greater tumor burden and less ER/PR expression in the PST group than in the upfront surgery group. As a result, in the total population, the DFS is worse in PST than in upfront surgery. After balancing the clinicopathological characteristics and controlling the confounding factors, the DFS is significantly improved in the PST group, especially in those getting pCR after PST.

## Data Availability Statement

The original contributions presented in the study are included in the article/supplementary material. Further inquiries can be directed to the corresponding author.

## Author Contributions

XY and HY had full access to all of the data in the study and take responsibility for the integrity of the data and the accuracy of the data analysis. Concept and design: XY, CW, YZ, BM, and HY. Acquisition, analysis, or interpretation of data: XY, CW, YZ, JH, and XS. Drafting of the manuscript: XY, CW, and YZ. Critical revision of the manuscript: YD, LS, JL, YD, HX, LG, WF, CQ, DC, YY, and HY. Statistical analysis: YD, LS, JL, YD, HX, and LG. Obtained funding: XY, WF, and HY. All authors contributed to the article and approved the submitted version.

## Funding

This study was supported by the Science and Technology Project of Zhejiang Provincial Department of Health (2020365865, 2020KY062), Zhejiang Provincial Natural Science Foundation of China under Grants (No. LY18H160152, NO. LY18H160033) and Wenling social development science and technology project (2021S00047).

## Conflict of Interest

The authors declare that the research was conducted in the absence of any commercial or financial relationships that could be construed as a potential conflict of interest.​​
